# Mesenchymal stem cells are efficiently transduced with adenoviruses bearing type 35-derived fibers and the transduced cells with the IL-28A gene produces cytotoxicity to lung carcinoma cells co-cultured

**DOI:** 10.1186/1471-2407-14-713

**Published:** 2014-09-25

**Authors:** Takeo Suzuki, Kiyoko Kawamura, Quanhai Li, Shinya Okamoto, Yuji Tada, Koichiro Tatsumi, Hideaki Shimada, Kenzo Hiroshima, Naoto Yamaguchi, Masatoshi Tagawa

**Affiliations:** Department of Molecular Cell Biology, Graduate School of Pharmaceutical Sciences, Chiba University, Chiba, Japan; Division of Pathology and Cell Therapy, Chiba Cancer Center Research Institute, Chiba, Japan; Department of Molecular Biology and Oncology, Graduate School of Medicine, Chiba University, Chiba, Japan; Department of Respirology, Graduate School of Medicine, Chiba University, Chiba, Japan; Department of Surgery, School of Medicine, Toho University, Tokyo, Japan; Department of Pathology, Tokyo Women’s Medical University Yachiyo Medical Center, Yachiyo, Japan

**Keywords:** Mesenchymal stem cells, Adenovirus, Type 35 adenovirus fiber, IL-28A

## Abstract

**Background:**

Transduction of human mesenchymal stem cells (MSCs) with type 5 adenoviruses (Ad5) is limited in the efficacy because of the poor expression level of the coxsackie adenovirus receptor (CAR) molecules. We examined a possible improvement of Ad-mediated gene transfer in MSCs by substituting the fiber region of type 5 Ad with that of type 35 Ad.

**Methods:**

Expression levels of CAR and CD46 molecules, which are the major receptors for type 5 and type 35 Ad, respectively, were assayed with flow cytometry. We constructed vectors expressing the *green fluorescent protein* gene with Ad5 or modified Ad5 bearing the type 35 fiber region (AdF35), and examined the infectivity to MSCs with flow cytometry. We investigated anti-tumor effects of MSCs transduced with *interleukin (IL)-28A* gene on human lung carcinoma cells with a colorimetric assay. Expression of IL-28A receptors was tested with the polymerase chain reaction. A promoter activity of transcriptional regulatory regions in MSCs was determined with a luciferase assay and a tumor growth-promoting ability of MSCs was tested with co-injection of human tumor cells in nude mice.

**Results:**

MSCs expressed CD46 but scarcely CAR molecules, and subsequently were transduced with AdF35 but not with Ad5. Growth of MSCs transduced with the *IL-28A* gene remained the same as that of untransduced cells since MSCs were negative for the IL-28A receptors. The *IL-28A*-transduced MSCs however suppressed growth of lung carcinoma cells co-cultured, whereas MSCs transduced with AdF35 expressing the *β-galactosidase* gene did not. A regulatory region of the *cyclooygenase-2* gene possessed transcriptional activities greater than other tumor promoters but less than the cytomegalovirus promoter, and MSCs themselves did not support tumor growth *in vivo*.

**Conclusions:**

AdF35 is a suitable vector to transduce MSCs that are resistant to Ad5-mediated gene transfer. MSCs infected with AdF35 that activate an exogenous gene by the cytomegalovirus promoter can be a vehicle to deliver the gene product to targeted cells.

## Background

Bone marrow-derived mesenchymal stem cells (MSCs) have ability to differentiate into many kinds of tissues under a certain condition [1, 2]. The pluripotency as progenitor cells indicates a potential clinical utility in multiple areas including tissue engineering. Furthermore, MSCs tend to migrate into inflammatory regions, damaged areas and tumors [3, 4], which increases applications of MSCs as a tool to deliver an agent to target tissues and cells. Gene and cell therapy can be one of the directions to use MSCs as a cellular vehicle that distributes a therapeutic gene product into target cells and tumors. Administration of transduced MSCs in the vicinity of tumors can transport the gene product into the microenvironment as well.

Adenoviruses (Ad)-mediated transduction is one of the efficient methods to transfer an exogenous gene into human cells. The transduction efficacy with Ad vectors is however influenced by expression levels of the receptor molecules on target cells [5]. Attachment of type 5 Ad (Ad5), commonly used in a gene transfer system, to cells is mediated primarily by the binding of Ad fibers, which include the shaft and the knob regions, to the cellular receptor, the coxsackie adenovirus receptor (CAR) molecules, and secondly by the interaction between Ad penton bases and integrin molecules [6]. Expression levels on CAR molecules are dependent on respective cells and are often down-regulated in human tumors, which resulted in poor transduction efficacy in CAR-low cells [7]. On the other hand, subgroup B Ad such as type 35 use CD46 molecules as one of the major receptors and infect cells in a CAR-independent manner [8]. CD46 is expressed in a variety of human cells and the expression levels were not down-regulated in tumors. Recombinant Ad5 of which the fiber region is replaced with that of type 35 Ad (AdF35) can therefore infect cells in a similar manner as type 35 Ad through the type 35-derived fibers, which may widen a scope of target cells that are restricted by non-ubiquitous CAR distributions.

A new class of interferon (IFN), type III IFNs comprising of IFN-λ1, -λ2 and -λ3 which are also known as interleukin-29 (IL-29), IL-28A and IL-28B, respectively, has a similar biological functions as type I IFNs such as IFN-α and IFN-β [9, 10]. The receptor complex of type III IFNs is composed of the IL-10 receptor beta (IL-10Rβ) and a novel IL-28 receptor alpha (IL-28Rα). In contrast to ubiquitous expression of IL-10Rβ, IL-28Rα expression is restricted to be tissue-specific, which subsequently confines the biological activities in IL-28Rα positive cells. The type III IFNs produce an anti-proliferative activity and induce apoptosis to a certain type of the receptor positive tumors including lung carcinoma [11] and esophageal carcinoma [12]. Moreover, several studies demonstrated that type III IFNs expressed in tumors achieved anti-tumor effects *in vivo* and some of the effects were mediated by non-immune mechanisms including anti-angiogenesis and by immunological responses such as activation of natural killer cells and dendritic cells [13–17].

In this study, we examined infectivity of Ad5 and AdF35 to human MSCs and investigated a possible use of MSCs as a vehicle to deliver gene products to tumors. We transduced MSCs with the *IL-28A* gene using a replication-incompetent AdF35 vector and tested whether the transduced MSCs produced cytotoxicty to tumor cells co-cultured. We also examined promoter activities in MSCs regarding transcriptional regulatory regions of the genes which are preferentially activated in human tumors.

## Methods

### Cells and mice

Human embryonic kidney HEK293 cells, human esophageal carcinoma YES-2 and TE-11 cells, human lung carcinoma OBA-LK1 cells, human immortalized fibroblasts OUMS-24 [18] and HFF cells [19], were cultured with RPMI1640 cells supplemented with 10% fetal bovine serum. MSCs derived from human bone marrow (PT-2501) (Cambrex, Rutherford, NJ, USA) were maintained with Mesenchymal Stem Cell Basal Medium (MSCBM; Cambrex). BALB/c *nu/nu* mice were purchased from Japan SLC (Hamamatsu, Japan).

### Flow cytometry for receptor expression

Cells were stained with fluorescein isothiocyanate (FITC)-conjugated anti-CD46 antibody (Ab) (BD Bioscience, San Jose, CA) or FITC-conjugated isotype-matched control Ab (BD Biosciences) as a control, or were reacted with anti-CAR (Upstate, Lake Placid, NY, USA), anti-CD51 (Chemicon, Temecula, CA, USA), anti-αvβ3 (Chemicon) or anti-αvβ5 Ab (Abcam, Cambridge, MA, USA) followed by FITC-conjugated goat anti-mouse IgG Ab (Kirkegaard & Perry, Gaithersburg, MD, USA). They were then analyzed for the fluorescence intensity with FACSCalibur (BD Bioscience) and CellQuest software (BD Bioscience).

### Construction of Ad vector

The *green fluorescent protein* (GFP), the *β-galactosidase* (LacZ), the human *IL-28A* genes were cloned into pShuttle 2 (Takara Bio, Tokyo, Japan) and then ligated with Adeno-X vector (Takara Bio) of which the fiber region was replaced with that of type 35 Ad. The fiber modified Ad DNA was produced by inserting the Eco RI fragment containing the type 35 Ad fiber region (Avior therapeutics, Seattle, WA) (AY271307 at 30827–33609) into the corresponding site of Adeno-X vector DNA. The fiber modified Ad expressing the above genes, AdF35-GFP, AdF35-LacZ, and AdF35-IL-28A, and type 5 Ad bearing the GFP gene (Ad5-GFP) were produced by transfecting the respective DNA into HEK293 cells and purified with an Adeno-X virus purification kit (BD Biosciences).

### Infectivity of Ad

Cells were infected with Ad5-GFP or AdF35-GFP at multiplicity of infection (MOI) of 3 or 30 for 30 min and were washed to remove Ad. Infected cells were cultured for 2 days and then analyzed for percentages of GFP-positive cells with FACSCalibur and CellQuest software. Cells of which fluorescence was greater than the brightest 5% of uninfected cells were judged as positively stained.

### Reverse transcription-polymerase chain reaction (RT-PCR)

First-strand cDNA was synthesized with Superscript III reverse transcriptase (Invitrogen, Carlsbad, CA) and amplification of equal amounts of the cDNA was performed with the following primers and conditions: for the *IL-28Rα* gene, 5’-GGGAACCAAGGAGCTGCTATG-3’ (sense) and 5’-TGGCACTGAGGCAGTGGTGTT-3’ (anti-sense), and 10 sec at 94°C for denature/20 sec at 58°C for annealing/28 cycles; for the *IL-10Rβ* gene, 5’-TATTGGACCCCCTGGAAT-3’ (sense) and 5’-GTAAACGCACCACAGCAA-3’ (anti-sense), and 10 sec at 94°C/20 sec at 50°C/28 cycles; for the *glyceraldehyde-3-phosphate dehydrogenase* (*GAPDH*) gene, 5’-ACCACAGTCCATGCCATCAC-3’ (sense) and 5’-TCCACCACCCTGTTGCTGTA-3’ (anti-sense), and 15 sec at 94°C/15 sec at 60°C/25 cycles.

### Cytotoxic test and enzyme-linked immunosorbent assay (ELISA)

OBA-LK1 cells were cultured in 96-well plates with MSCs uninfected or infected with AdF35-IL-28A or AdF35-LacZ (MOI = 100), at a ratio of 10: 1 or 10: 3. Cell viabilities after 4-days culture were assayed with a WST kit (Dojindo, Kumamoto, Japan) which detected the amounts of formazan produced from the WST-8 reagent with the absorbance at 450 nm (WST assay). OBA-LK1 cells were also labeled with PKH 26 dye according to the manufacturer’s protocol (Sigma-Aldrich, St Louis, MO, USA) and cultured with MSCs, uninfected or infected with AdF35-IL-28A or AdF35-LacZ (MOI = 100), at a ratio of 10: 1 or 10: 3 for 4 days. They were then stained with Hoechst 33342 dye (Molecular Probes, Eugene, OR, USA) and numbers of PKH 26 positive and Hoechst 33342 positive cells were counted with confocal microscope (Olympus, Tokyo, Japan). The amounts of secreted IFN-28A were determined by an ELISA kit (R&D Systems, Minneapolis, MN).

### Dual luciferase assay

Genomic fragments containing a transcriptional regulatory region of the *midkine* (0.6 kb, GenBank: D10604) [20], the *survivin* (0.5 kb, GenBank: U75285) [21], or the *cyclooxygenase-2* (0.3 kb, GenBank: U04636) gene [22] were cloned into pGL-2 basic vector (Promega, Madison, WI, USA) that contained the *firefly luciferase* gene. Plasmid DNA containing the respective genomic fragments, pGL-control vector (Promega) harboring the SV40 T antigen promoter-linked *firefly luciferase* gene, pGL-2 basic vector containing the cytomegalovirus (CMV) promoter or pGL-basic vector without any transcriptional regulatory regions (Promega), and a control vector, the *renilla luciferase* gene fused with the *herpes simplex virus-thymidine kinase* gene promoter (pRL-TK, Promega), at a molar ratio of 10: 1, was transfected into MSCs with a lipofectin reagent (Life Technologies, Gaithersburg). Cell lysate on day 2 was assayed for the luciferase activity with the dual luciferase reporter assay (Promega). The firefly luciferase activity was standardized by the amounts of luminescence produced by renilla luciferase and the relative activity was expressed as a percentage of the SV40 T antigen promoter-mediated activity.

### Animal study

YES-2 cells (1 × 10^6^) and MSCs or OUMS-24 cells at a ratio 5: 1 or 2: 1 (2 × 10^5^ or 5 × 10^5^) were inoculated subcutaneously into BALB/c *nu*/*nu* mice (6-week-old females). Tumor volume was calculated according to the formula (1/2 × length x width^2^).

### Statistical analysis

Statistical analysis was conducted with the one-way analysis of variance (ANOVA).

## Results

### Expression of Ad receptors on MSCs

**Figure 1:**
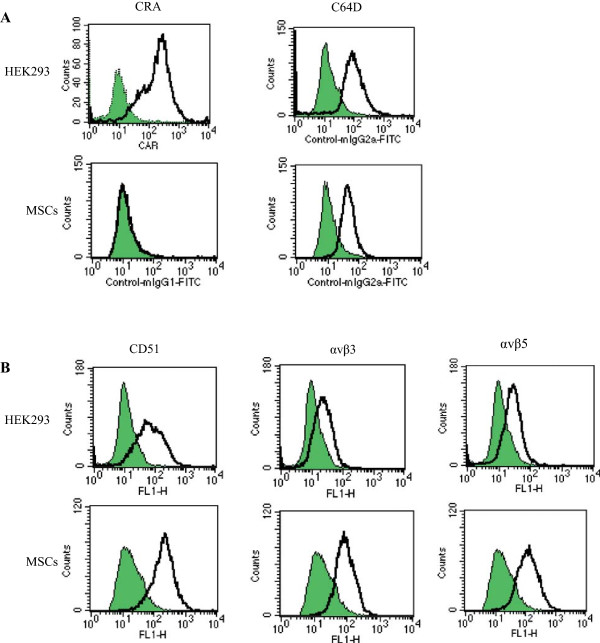
**Expression of Ad receptors on HEK293 cells and MSCs.** Representative flow cytometry profiles of HEK293 cells and MSCs that were stained with **(A)** anti-CAR, anti-CD46, **(B)** anti-CD51, anti-integrin αvβ3 or anti-integrin αvβ5 Ab. Shaded areas and bold lines show unstained and stained cells, respectively.

**Figure 2:**
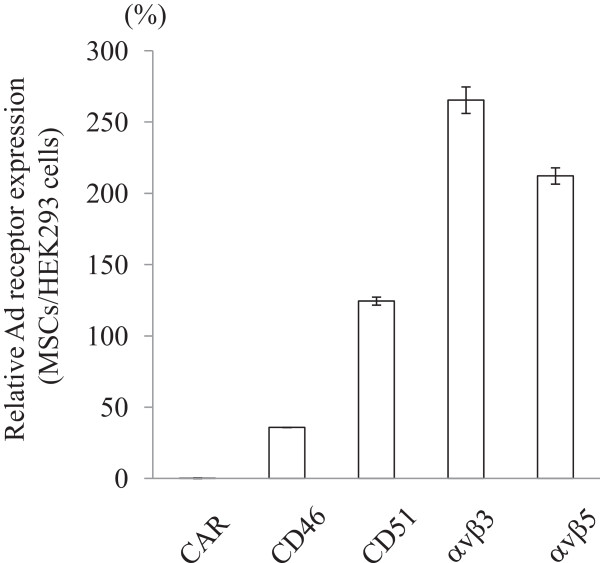
**Compared expression levels of Ad receptors between MSCs and HEK293 cells.** Mean fluorescence intensity in staining profiles was expressed as an arbitrary FL1 unit and the relative ratios, MSCs/HEK293 cells, are shown as a percentage. SE bars are also shown (n = 3).

We examined expression levels of CAR and CD46 molecules, the major Ad receptors of type 5 and type 35, respectively, on HEK293 cells and MSCs (Figure 1A). HEK293 cells, often used for Ad productions, expressed both receptors, whereas MSCs scarcely expressed CAR but were positive for CD46 expression. We also tested integrin molecules which were subsidiary receptors for type 5 Ad (Figure 1B). Both HEK293 cells and MSCs expressed CD51 that corresponded to integrin αv chain, αvβ3 and αvβ5 molecules. We calculated relative expression levels of these receptor molecules on MSCs in comparison with HEK293 cells and showed that MSCs expressed poorly CAR, moderately CD46 and well integrin molecules (Figure 2).

### Infectivity of Ad5 and AdF35 to MSCs

**Figure 3:**
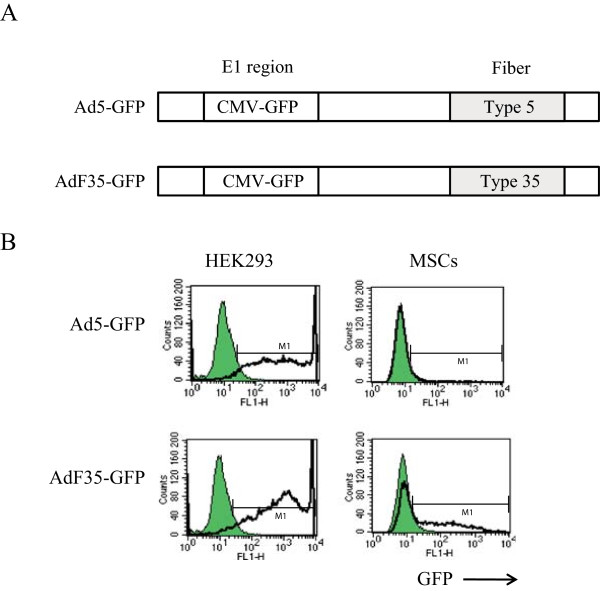
**Infectivity of Ad to MSCs. (A)** Schematic structures of Ad5-GFP and AdF35-GFP. The E1 region was replaced with the CMV promoter-linked GFP gene. **(B)** Representative flow cytometry profiles of HEK293 cells and MSCs that were infected with Ad5-GFP or AdF35-GFP. M1 indicates positively stained population, and shaded areas and bold lines show uninfected and infected cells, respectively.

**Figure 4:**
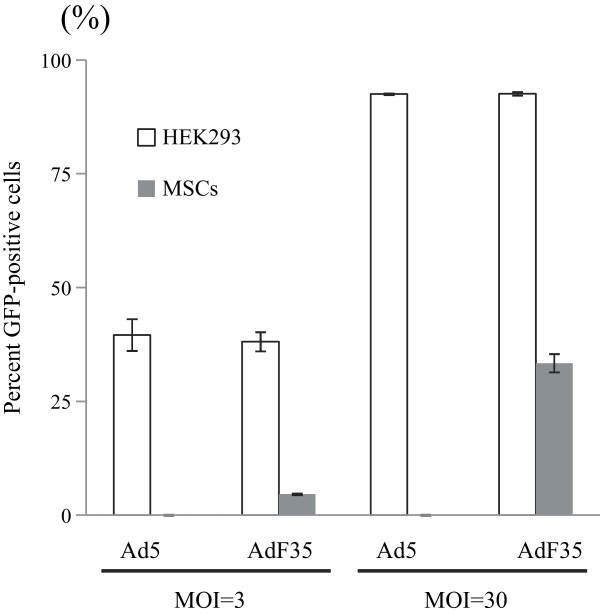
**Infectivity of Ad5 and AdF35 to MSCs.** MSCs or HEK293 cells were infected with Ad5-GFP or AdF35-GFP (MOI = 3 or 30) and cell populations greater than the brightest 5% of uninfected cells were judged as GFP-positive. SE bars are also shown (n = 3).

We investigated efficacy of Ad5- and AdF35-mediated transduction with respective Ad bearing the *GFP* gene (Figure 3A). HEK293 cells became GFP positive after transduction with either Ad5-GFP or AdF35-GFP, but MSCs expressed GFP only when transduced with AdF35-GFP (Figure 3B). Percentages of GFP positive HEK293 cells were not different when they were transduced either with Ad5-GFP or AdF35-GFP (Figure 4). In contrast, GFP positive MSCs cells were undetected with Ad5-mediated transduction and the positive percentages after transduction with AdF35-GFP were lower than those of HEK293 cells. The differential GFP positive rates were attributable to viral infectivity to the cells since both Ad5-GFP and AdF35-GFP used the same CMV promoter.

### Anti-tumor effects of MSCs infected with AdF35-IL-28A

**Figure 5:**
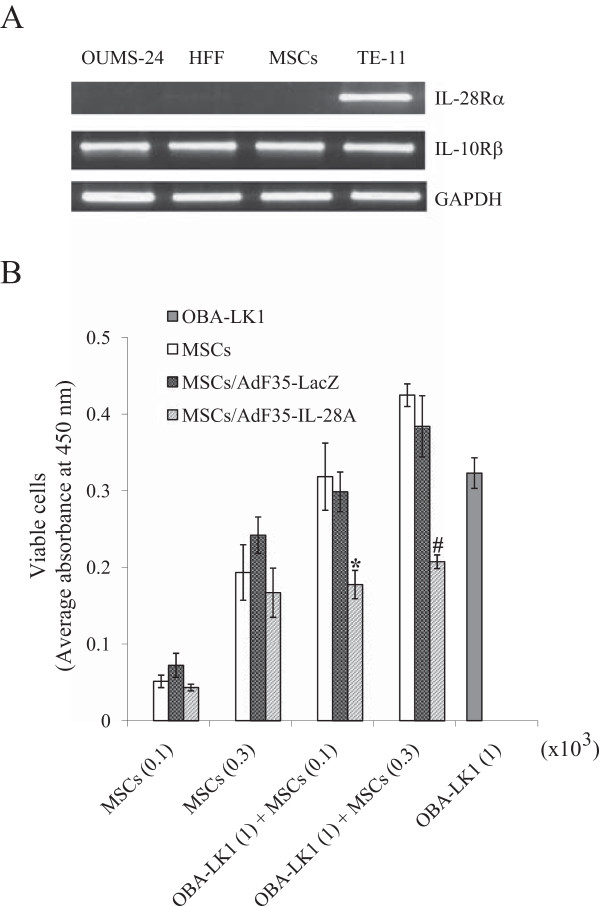
**Growth inhibition of MSCs transduced with AdF35-IL-28. (A)** Expression of IL-28 receptor complexes in human fibroblasts and MSCs. RT-PCR showed IL-28Rα and IL-10β gene products. TE-11 cells were used as a positive control for the gene expression and GAPDH as a loading control. **(B)** MSCs (100 or 300/well), uninfected or infected with AdF35-IL-28A or AdF35-LacZ (MOI = 100), were cultured alone or with OBA-LK1 cells (1,000/well) for 4 days. Viability of each group was detected with the WST-8 reagent and expressed as absorbance at 450 nm. SE bars are also shown (n = 3). *P < 0.05 and ^#^P < 0.01, comparing MSCs infected with AdF35-IL-28A versus MSCs uninfected or infected with AdF35-LacZ.

IL-28A produced cytotoxic effects on cells expressing the receptor complex, IL-28Rα and IL-10Rβ. We examined the expression on MSCs together with immortalized fibroblasts, OUMS-24 and HFF cells, and esophageal carcinoma TE-11 cells as a reference of normal cells and as a positive control for the IL-28A receptor complex, respectively (Figure 5A) [12]. MSCs and the fibroblasts expressed IL-10Rβ but not IL-28Rα, demonstrating that MSCs were insensitive to IL-28A. In contrast, the esophageal carcinoma cells were positive for both molecules [12].

We then examined possible cytotoxicity of IL-28A released from MSCs in a co-culture experiment. Lung carcinoma OBA-LK1 cells were positive for the IL-28A receptor complex and the growth was suppressed by recombinant IL-28A [11]. We infected MSCs with AdF35-IL-28A or AdF35-LacZ as a control and detected IL-28A released from MSCs with ELISA at 363 ± 4.61 pg/ml/day per 10^3^ MSCs. IL-28A-sensitive OBA-LK1 cells were mixed with the MSCs and the viable cell numbers were estimated with the WST assay (Figure 5B). Viability of MSCs that were either uninfected or infected with Ad-IL-28A or Ad-LacZ was not statistically different, showing that expression of IL-28A did not induce growth suppression in MSCs. In contrast, absorbance of a mixed population consisting of OBA-LK1 cells and AdF35-IL-28A-infected MSCs was lower than that of a mixture of OBA-LK1 cells and either uninfected MSCs or AdF35-LacZ-infected MSCs. Absorbance of the cell mixture of OBA-LK1 cells and AdF35-IL-28A-infected MSCs was even lower than that of OBA-LK1 cells alone, indicating that IL-28A released from MSCs inhibited growth of OBA-LK1 cells.

We confirmed growth inhibitory activities of MSCs transduced with AdF35-IL-28A in a different assay (Table 1). OBA-LK1 cells were labeled with PKH 26 and cultured with MSCs for 4 days. We then stained all the cells with Hoechst 33342 and calculated numbers of PKH 28 positive cells among Hoechst 33342 positive cells. Percentages of PKH 28 positive OBA-LK1 cells were about 80% because the PKH 28 labeling was not complete under the experimental condition. OBA-LK1 cells cultured with untransduced MSCs further decreased the PKH 28 positive ratio since PKH 26 negative MSCs were also counted. Percentages of PKH 26 positive cells in cell mixtures were not different between co-culture with uninfected MSCs and that with AdF35-LacZ-infected MSCs irrespective of a ratio of the mixtures. The percentages however significantly lower in co-culture with AdF35-IL-28A-infected MSCs compared with cell mixture with uninfected MSCs or AdF35-LacZ infected MSCs. These data demonstrated that MSCs-derived IL-28A inhibited growth of OBA-LK1 cells.

**Table 1:** **Growth suppression of OBA-LK1 cells cultured with transduced MSCs**

OBA-LK1	MSCs infected with	Mixed cell ratio (OBA-LK1 : MSCs)	PKH 26 positive cells (Percentage ± SE)^1^
(+)	(−)		80.2 ± 2.0
(+)	None	10 : 1	57.4 ± 4.2*
(+)	AdF35-LacZ	10 : 1	65.6 ± 4.1*
(+)	AdF35-IL28A	10 : 1	47.6 ± 2.7*
(+)	None	10 : 3	67.0 ± 4.4^#^
(+)	AdF35-LacZ	10 : 3	56.1 ± 2.0^#^
(+)	AdF35-IL-28A	10 : 3	26.4 ± 1.4^#^

OBA-LK1 cells (3.3 ×10^4^) stained with PKH 26 were cultured with MSCs at a ratio indicated, and all the cells were then stained with Hoechst 33342 on day 4. ^1^Percentages of PKH 26 positive cells among Hoechst 33342 positive cells and SEs are also shown (n = 3).

*P < 0.05 and ^#^P < 0.01, comparing between cells mixed with AdF35-IL-28A-infected MSCs and cells mixed with uninfected MSCs or AdF35-LacZ-infected MSCs.

### Transcriptional regulation and growth assistance in MSCs

We investigated whether a putative tumor promoter could activate the *luciferase* gene in non-tumorous MSCs. We therefore examined transcriptional regions of the *midkine*, the *survivin* and the *COX-2* genes for the promoter activity in MSCs with the SV40 T antigen promoter region as a reference (Table 2). These regions are often used for activation of a transgene in a tumor-specific manner. A regulatory region of the *COX-2* gene activated the *luciferase* gene greater than that of the *midkine* or the *survivin* gene. A transcriptional activity of the COX region was greater than that of the SV40 T antigen promoter, but much less than that of the CMV promoter which is commonly used for transgene activations in many cells.

**Figure 6:**
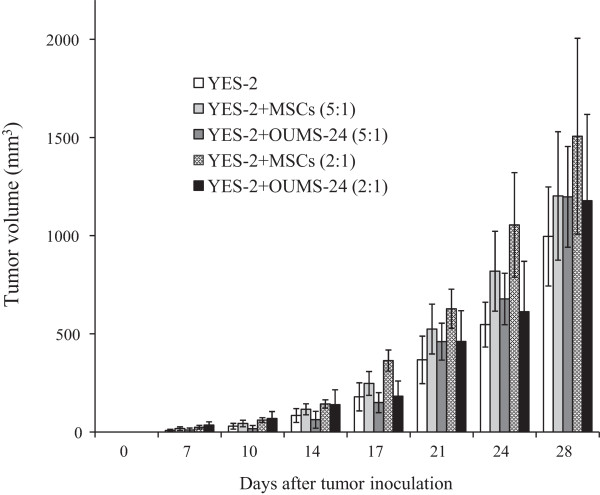
**MSCs did not influence growth of co-injected tumors.** YES-2 cells and MSCs or OUMS-24 cells were inoculated subcutaneously into BALB/c *nu*/*nu* mice at the ratios indicated. Average tumor volumes and SE bars are shown (n = 6).

We also examined a possible tumor growth-promoting activity of MSCs with animal experiments (Figure 6). We inoculated YES-2 esophageal carcinoma cells into nude mice without or with MSCs or fibroblasts OUMS-24. Tumor growth of mixed populations, irrespective of the ratio or mixed cells, was not different from that of YES-2 cells alone, demonstrating that MSCs, like fibroblasts, did not support tumor growth of YES-2 esophageal carcinoma cells co-injected.

**Table 2:** **Promoter activity of transcriptional regulatory regions in MSCs**

Transcriptional regulatory region	Luciferase activity (average ± SE)^1^
(−)	16.8 ± 8.9
SV40 T antigen	100.0 ± 11.5*
Midkine	66.8 ± 27.6*
Survivin	47.5 ± 14.7*
COX-2	368.2 ± 182.8*
CMV	6323.4 ± 2067.3*

^1^Relative luciferase activity was calculated based on the SV40 T antigen promoter-mediated activity as 100%. SEs are shown (n = 3).

*P < 0.05, comparing between the COX-2 region and either the SV40 T antigen, the survivin regulatory region, the midkine regulatory region or the CMV promoter.

## Discussion

We showed that MSCs were resistant to Ad5-mediated gene transfer but were transduced with fiber-modified AdF35 vectors. The transduction preference was linked with the Ad receptor expression on MSCs, which were negative for CAR but positive for CD46 molecules. Comparison of receptor expressions between HEK293 cells and MSCs further showed that CD46 expression levels on MSCs were not as great as those on HEK293 cells, which resulted in lower transduction efficacy of AdF35 to MSCs than to HEK293 cells. Interestingly, expression levels of αvβ3 and αvβ5 integrin molecules were greater on MSCs than on HEK293 cells. Nevertheless, transduction of MSCs with Ad5 was not detected, indicating that the integrin molecules did not play a role as an auxiliary receptor in MSCs although the integrin molecules were demonstrated to be the major receptor of Ad5 in CAR-deficient cells [23]. In addition, the integrin molecules were shown to enhance AdF35-mediated gene transduction [24], but the present study suggested that elevated expression levels of integrin molecules on MSCs cells could not restore transduction efficacy of AdF35 to MSCs to the same level as to HEK293 cells. Many factors seem to be involved in mechanisms underlying Ad infectivity and the mechanisms can be different among cell types tested. They may include a possible threshold level of the receptor expression necessary for Ad infection and a presumable reciprocal interaction among the receptor molecules. CD46 is not a sole receptor for AdF35 and the expression levels were relatively low in freshly isolated MSCs [25]. Nevertheless, the MSCs from adult donors were infected with AdF35 [25], and we presume that Ad vectors bearing the type 35 fiber is currently one of the efficient vectors for gene transfer into MSCs.

Usage of gene modified MSCs has several advantages over direct Ad administrations in the anti-tumor activity. Transduced MSCs, injected intratumorally, tend to localize at the tumor sites in contrast to Ad which are subjected to a rapid washout from the injection sites [26]. Propensity of MSCs to migrate into tumors is thus favorable for MSCs-mediated anti-tumor effects [27] although the property was dependent on an experimental system [28]. Administration of MSCs into a tumor site also needs careful consideration since MSCs can promote the tumor growth due to the ability to constitute and maintain the microenvironments around tumors. We thereby examined the possible growth-enhancing activity *in vivo* and showed that MSCs did not support tumor growth of esophageal carcinoma cells co-injected. MSCs were not able to enhance tumor growth in nude mice but the possibility of tumor promoting actions needs to be studied in different experimental models. On the other hand, the present study showed that untransduced MSCs did not achieve any anti-tumor effects by themselves. The growth suppressing activity of transduced MSCs was thus attributable to IL-28A since the suppression was dependent on cell numbers of *IL-28*-transduced but not on *β-galactosidase*-transduced MSCs. A property of expressed transgene products is also crucial for therapeutic efficacy of MSCs-mediated gene delivery. For example, IL-28A not only induces tumor cell death through apoptosis but activates inmate and acquired immunity through augmented natural killer activities and facilitated antigen presentation [12, 14–17]. Moreover, IL-28A in vivo influences and modulates tumor microenvironments such as inhibition of angiogenesis, which can be mediated by other cytokines [13].

Recently, several reports demonstrated anti-tumor effects produced by MSCs-mediated delivery of replication-competent Ad into tumors [28, 29] and a clinical research revealed benefits of such autologous MSCs in neuroblastoma patients [30]. Tumor cells were initially used for cell-mediated delivery of replication-competent Ad [31] since tumor cells well supported Ad replication compared with non-transformed cells. MSCs may not be effective in the light of production of progenitor Ad because of the low proliferation rate. We examined the promoter activity of transcriptional regulatory regions which activated the *Ad E1A* gene and subsequently enabled Ad replication-competent within tumors [32–34]. The *COX-2* region gave greater activities than the *midkine* or the *survivin* region. Expression levels of midkine and survivin in adult tissues are often associated with proliferation rates of cells [35] and relatively low promoter activities of these regions could reflect the low growth rates of MSCs. Instead, COX-2 expression can be liked with inflammatory responses [36, 37]. Elevated promoter activity of the *COX-2* region in MSCs may be related to MSCs’ propensity to migrate toward inflammatory sites. Nevertheless, a promoter activity of the *COX-2* region was much lower than that of the CMV promoter which is commonly used to activate a transgene in replication-incompetent Ad. These data suggest that MSCs are a suitable cell-mediate vehicle for CMV promoter-driven replication-incompetent Ad rather than for replication-competent Ad in which the *E1A* is activated by an exogenous transcriptional regulatory region.

## Conclusions

We demonstrated anti-tumor effects of MSCs-mediated delivery of IL-28A to lung carcinoma cells in the vicinity. A local administration of gene-modified MSCs can deliver the gene product to targets and is one of the cell therapies for cancer. AdF35 is a better vector than conventional type 5 Ad in transducing MSCs because of its enhanced infectivity. Immune responses against gene-modified MSCs are less significant as long as MSCs are autologously isolated. Nevertheless, when MSCs are infected with replication-competent Ad, cell-mediated immunity against viral gene-loaded MSCs will be generated. Further investigations are required for the cell-mediated immunity in the light of Ad-loaded MSCs and for the strategy to evade host immunity such as use of immunosuppressive agents.

## Abbreviations

MSCs: Mesenchymal stem cells
Ad: Adenoviruses
CAR: Coxsackie adenovirus receptor
IFN: Interferon
IL: Interleukin
FITC: Fluorescein isothiocyanate
Ab: Antibody
GFP: Green fluorescent protein
LacZ: β-galactosidase
MOI: Multiplicity of infection
RT-PCR: Reverse transcription-polymerase chain reaction
GAPDH: Glyceraldehyde-3-phosphate dehydrogenase
ELISA: Enzyme-linked immunosorbent assay
CMV: Cytomegalovirus.
